# Gastric Hyperplastic Polyp Causing Upper Gastrointestinal Hemorrhage and Severe Anemia in a Dog

**DOI:** 10.3390/vetsci9120680

**Published:** 2022-12-07

**Authors:** Kihoon Kim, Binwon Jun, Sangwoo Han, Daseul Kim, Hyungjun Kim, Hyosung Kim, Sunhee Do, Jaehwan Kim, Hwiyool Kim, Seunghwa Yang

**Affiliations:** 1 Department of Veterinary Surgery, College of Veterinary Medicine, Konkuk University, Seoul 05029, Republic of Korea; 2 Department of Surgery, Dr. Dog Animal Medical Center, Goyang 10406, Republic of Korea; 3 Department of Internal Medicine, Dr. Dog Animal Medical Center, Goyang 10406, Republic of Korea; 4 Department of Veterinary Clinical Pathology, College of Veterinary Medicine, Konkuk University, Seoul 05029, Republic of Korea; 5 Department of Veterinary Medical Imaging, College of Veterinary Medicine, Konkuk University, Seoul 05029, Republic of Korea

**Keywords:** anemia, dog, hyperplastic polyp, stomach

## Abstract

**Simple Summary:**

The current paper reports a rare case of gastric hyperplastic polyp in a dog with upper gastrointestinal hemorrhage, resulting in severe anemia. Gastric hyperplastic polyps are generally benign and found incidentally during endoscopic examination, or necropsy. Although they are asymptomatic, depending on their size or location, they can sometimes result in clinical signs including chronic occult blood loss, abdominal pain, or gastric tract obstruction. In human medicine, gastric hyperplastic polyps causing acute blood loss anemia have rarely been reported. However, there is no such case reported in veterinary medicine so far. To our knowledge, this is the first report describing a gastric hyperplastic polyp that caused severe anemia because of acute blood loss in a dog.

**Abstract:**

An 11-year-old castrated male Shih Tzu was referred for lethargy and melena. The hematocrit level was 18.8% (normal range: 36–56%), indicating severe anemia. Abdominal ultrasound revealed a round-to-oval-shaped mass in the stomach. Computed tomography (CT) revealed an intraluminal mass (17 × 12 × 15 mm) cranial to the pyloric antrum. After obtaining informed consent from the owner, exploratory laparotomy and subsequent gastrostomy were performed, showing an ulcerated mass potentially responsible for the severe anemia. A lump of hair was firmly attached to the ulcerated surface of the mass. After complete removal of the mass, the anemia resolved spontaneously. Histological examination revealed that the mass was a gastric hyperplastic polyp. At the 6-month follow-up, the dog was healthy with a normal hematocrit level. Gastric hyperplastic polyps are tumor-like lesions arising from the mucosal surface of the stomach, and projecting into the lumen. They can appear in any part of the stomach, and are usually found incidentally during gastric endoscopy or necropsy. The clinical signs include chronic occult blood loss, abdominal pain, and gastric tract obstruction. Gastric polyps causing acute blood loss anemia have rarely been reported in human medicine. To our knowledge, this is the first report describing a gastric hyperplastic polyp that caused severe anemia because of acute blood loss in a dog.

## 1. Introduction

Gastric polyps are tumor-like lesions arising from the mucosa of the stomach and projecting into the gastric lumen, that can be either non-neoplastic or neoplastic lesions [1,2,3,4]. The pathogenesis of gastric polyp in dogs is still unknown [5,6]. Compared with humans, gastric polyps appear to be less common in dogs and cats, and are usually found incidentally during gastric endoscopy or necropsy [1,5]. Although they are commonly asymptomatic, large hyperplastic polyps may cause chronic occult blood loss, abdominal pain, or gastric tract obstruction [7].

Gastric polyps can occur in any part of the stomach [2,8]. They are generally either sessile or pedunculated, measuring about 7–60 mm [1,9]. Generally, polypoid non-neoplastic growths are classified as either hyperplastic (regenerative) or inflammatory (benign lymphoid), while polyps with neoplastic features are either adenomas or carcinomas [10]. However, according to the WHO classification of tumors in the alimentary system of domestic animals, only two entities are recognized: hyperplastic, and inflammatory gastric polyps [11]. Hyperplastic polyps are epithelial lesions with elongated or distorted foveolae and cystically dilated glands, identical to human lesions, while inflammatory polyps have a granulation core tissue with infiltration of various inflammatory cells [4,11]. The malignant potential of gastric hyperplastic polyp in veterinary medicine has not been reported thus far.

There are several case reports describing gastric hyperplastic polyp causing acute bleeding in the human literature [7,8,12]. However, to the best of our knowledge, no such cases have been reported yet in veterinary medicine. The aim of this paper is to report a gastric hyperplastic polyp causing severe anemia in a dog, that spontaneously resolved after polyp removal.

## 2. Case Presentation

An 11-year-old castrated male Shih Tzu was referred for lethargy and melena. On physical examination, the color of the mucous membrane was pale, and the capillary refill time was 2 s. A complete blood count revealed that the white blood cell count was 23,650/µL (normal range: 6000–17,000/µL); hematocrit (HCT) level, 18.8% (normal range: 36–56%); hemoglobin level, 8.7 g/dL (normal range: 11.0–19.0 g/dL); and platelet count, 424,000/µL (normal range: 117,000–460,000/µL). Blood chemistry analysis showed an alkaline phosphatase level of 275 U/L (normal range: 12–122 U/L). Blood samples were collected and submitted for the anemia panel, which showed negative findings.

**Figure 1:**
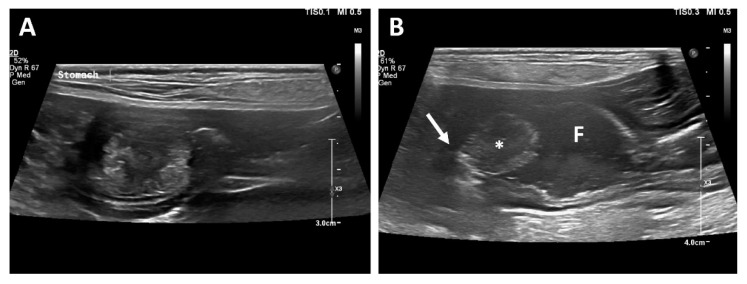
Longitudinal ultrasonographic examination. A mass arising from the mucosal layer was identified (**A**). A sessile echogenic lesion (asterisk) with a broad base (arrow) arising from the mucosa was identified after feeding water (**B**). Fluid (F) is present in the stomach.

Thoracic and abdominal radiography revealed no significant findings. Abdominal ultrasonographic examination (EPIQ 5G; Philips Ultrasound, Bothell, WA, USA) revealed a solitary and round-to-oval-shaped intraluminal mass cranial to the pyloric antrum (Figure 1). In order to obtain information regarding the base of the mass, a hydrogram was obtained after feeding 500 mL of water. The mass was assumed to be sessile because its base was wide, and it showed little or no displacement during peristalsis. The patient’s gastric motility was normal, and the lesion showed mixed echogenicity.

**Figure 2:**
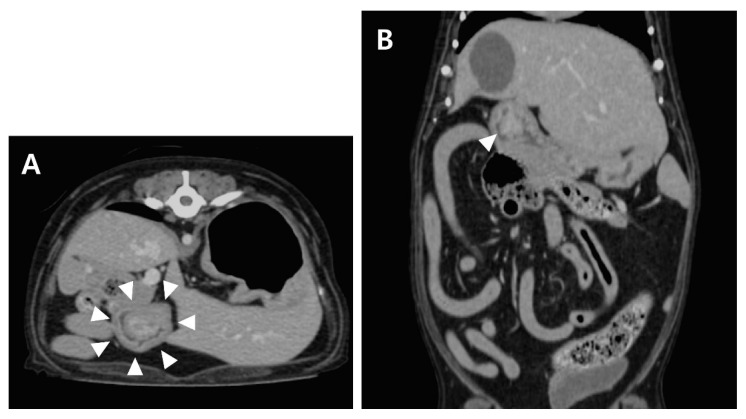
Post-contrast transverse (**A**) and dorsal (**B**) CT images of the abdomen. A wide-based, polypoid mass (arrowhead) with heterogeneous contrast enhancement.

Computed tomography (CT; LightSpeed, GE Medical Systems, Milwaukee, WI, USA) was performed to evaluate the gastric lesion. With the patient positioned in ventral recumbency, a contrast study was performed after administration of the contrast medium (Omnipaque 300, GE Healthcare, Milwaukee, WI, USA). Contrast uptake was assessed in the late venous phase. Post-contrast CT images revealed a mass (17 × 12 × 15 mm) cranial to the pyloric antrum, and the contrast enhancement of the mass was similar to that of the surrounding gastric wall (Figure 2). No contrast leakage was identified.

**Figure 3:**
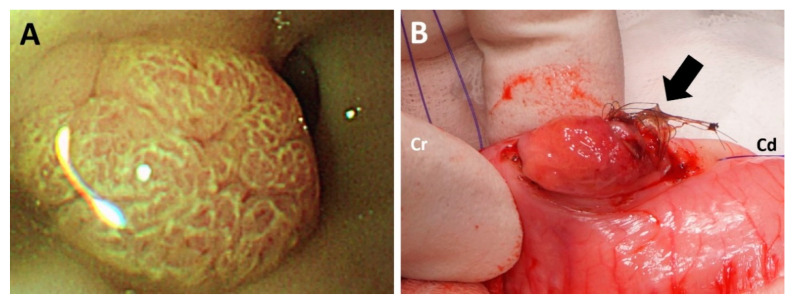
Gross appearance of the cranial surface of the mass during endoscopy (**A**). A lump of hair firmly attached to the ulcerated surface was identified at the caudal side of the mass (**B**).

Endoscopy (EVIS CV-260; Olympus, Center Valley, PA, USA) was performed to further observe gross appearance of the mass in the stomach. The mass was located cranially to the pyloric region (Figure 3A).

Immediate blood transfusion was performed at the time of presentation, and the HCT level subsequently increased to 35.3%. However, the HCT level decreased to 28.7% two days after blood transfusion. Although bleeding was not identified, exploratory laparotomy was performed with informed consent from the owner.

Preanesthetic agents, including butorphanol (0.5 mg/kg, Butophan; Myungmoon Pharmaceutical, Seoul, Korea) and midazolam (0.1 mg/kg, Midazolam; Bukwang Pharmaceutical, Seoul, Korea), were administered intravenously. After administration of propofol (6 mg/kg, Provive Inj.; Myungmoon Pharmaceutical, Seoul, Korea), inhalation anesthesia using isoflurane (Isoflurane; Choongwae Pharmaceutical, Seoul, Korea) was maintained during surgery.

With the patient in dorsal recumbency, the skin and abdominal wall were incised routinely. The mass within the stomach was palpated cranial to the pyloric antrum. Gastrostomy was subsequently performed, and the mass was identified on the dorsal wall of the gastric body. No source of bleeding was found at the cranial surface of the mass, as identified during endoscopy. However, in contrast to the cranial surface of the mass, the caudal surface of the mass was ulcerated with hemorrhage, which was considered to be a bleeding focus. Moreover, a lump of hair was firmly attached to the mass (Figure 3B). The mass and adjacent mucosa were completely resected with a margin of 2 cm. The abdomen was lavaged with warm saline several times prior to closure. The abdominal wall and skin were closed routinely. Recovery from anesthesia was uneventful.

The hematocrit level of the patient increased spontaneously, reaching 32.9% on the ninth day after surgery, and 35.5% on the twelfth day. The dog was discharged on the twelfth day after surgery. At the 2-month follow-up, the dog showed unremarkable recovery. At the 6-month follow-up, the hematocrit level was 44.0%.

**Figure 4:**
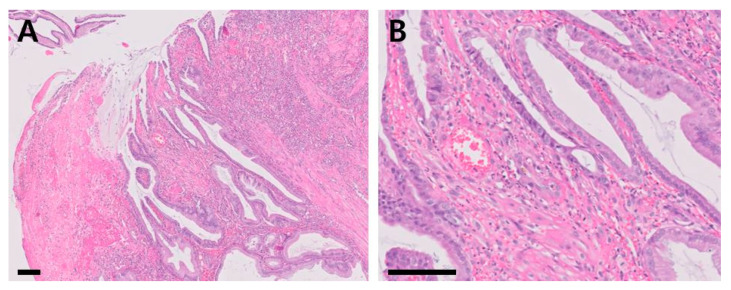
Histopathological sections of the mass (hematoxylin and eosin stain; (**A**): ×100, scale bar = 100 µm, (**B**): ×400, scale bar = 50 µm).

Histologic examination (Figure 4) revealed that the mucosa was expanded by a polypoid, moderately cellular neoplasm consisting of epithelial cells that lined regular and irregular tubulopapillary structures, supported by lamina proprial fibrovascular, and muscular stromal cores. Peripheral to the polypoid lesion, the remaining pyloric mucosal epithelium was mildly to moderately hyperplastic, with cryptal elongation and mildly increased tortuosity (Figure 4A). The epithelial cells were cuboidal-to-columnar, and had moderate amounts of bright eosinophilic, vacuolated cytoplasm with distinct cellular borders. The nuclei were round, oval, elongated and paracentric or basal, with stippled chromatin, and a single, small, deeply eosinophilic nucleolus. Mild anisocytosis and mild-to-moderate anisokaryosis were observed (Figure 4B). No microorganisms were identified in the histologic section and the Warthin-Starry silver staining result was negative (Supplementary Material Figure S1). The final diagnosis of the mass was hyperplastic polyp.

## 3. Discussion

Gastric polyps are defined as lesions protruding from the mucosal layer into the lumen of the stomach [10]. These polyps are rarely encountered in dogs, but may be incidentally detected during endoscopy of the upper gastrointestinal tract, or postmortem examination [1,13]. The exact etiology or pathogenesis of gastric polyps in dogs remains unclear [6]. According to the human literature, they are assumed to be associated with *Helicobacter (H) pylori* in more than 90% of patients [14]. Thus, some researchers have concluded that *H. pylori* eradication might be attributed to complete resolution or significant size reduction, although the exact role of *H. pylori* in the formation of gastric polyps is unclear [1,10]. In this case report, microorganisms were not identified by histological examination. Moreover, the Warthin-Starry silver stain, a special staining technique for spirochetes including *H. pylori*, revealed a negative result. Gastric hyperplastic polyps occur secondary to inflammatory responses, such as gastritis, and result from an exaggerated mucosal response to injury [4]. Since the hair was attached firmly to the mass, the polyp might have resulted from mucosal injury secondary to the lump of hair. However, considering the possibility of incidental coincidence of the polyp and the lump of hair, the exact reason for polyp formation remains unclear.

Gastric polyps are usually asymptomatic. However, depending on their size or location, they can result in clinical signs, including vomiting or intermittent gastrointestinal obstruction [13]. Antral or pyloric polypoid lesions of significant size, that cause intermittent gastric outlet obstruction, have been reported in dogs [3,9]. In humans, in addition to gastrointestinal obstruction, gastric hyperplastic polyps with bleeding that cause acute anemia have been reported. These cases present with clinical signs of melena, indicating upper gastrointestinal hemorrhage [7,8,12]. In the case reported here, because the dog showed melena, hemorrhage from the gastrointestinal tract was suspected. On the basis of the radiographic, ultrasonographic and CT findings, and the ulcerated appearance of the polyp during surgery, since the gastric polyp was the only malformation in the entire gastrointestinal tract, it was suspected to be the cause of anemia. In addition, since the clinical signs of anemia and melena spontaneously resolved after removal of the gastric polyp, the gastric hyperplastic polyp was highly assumed to be the reason for gastrointestinal hemorrhage causing severe anemia. To the best of our knowledge, this is the first case report describing a gastric hyperplastic polyp that caused severe anemia due to acute blood loss in a dog.

Biologically, gastric polyps are classified as neoplastic or non-neoplastic. For domestic animals, the World Health Organization classifies non-neoplastic polypoid growth into two entities: hyperplastic, and inflammatory polyps. Gastric hyperplastic polyps are typically observed in the antrum of the stomach. Histologically, they consist of hyperplastic, elongated, and distorted pits extending deep into the stoma and containing pyloric glands, chief cells, or parietal cells. On the other hand, inflammatory polyps are characterized by the presence of a normal epithelium lining a granulation tissue infiltrated by mixed inflammatory cells, including lymphocytes, or plasma cells. They are also sometimes accompanied by lymphoid aggregates with germinal cores [1,8,11].

Studies describing cases in human patients suggested that atypical surface morphology and bleeding in gastric hyperplastic polyps may be associated with malignant changes [12]. However, compared to human medicine, the majority of gastric hyperplastic polyps in veterinary medicine have been benign, and did not exhibit morphological or molecular changes suggestive of malignancy. Previously, Amorim et al. demonstrated no convincing relationships between possible risk factors, and gastric hyperplastic polyps [4].

Surgical treatment depends on the type and location of the polyps in the stomach [9]. Endoscopic removal is a common approach used for pedunculated polyps. However, endoscopic polypectomy is not recommended for sessile polyps with a broad base [3], since endoscopic polypectomy, in such cases, is associated with complications such as incomplete resection of the lesion, or gastrointestinal perforation [5,15]. In human medicine, recurrence of gastric hyperplastic polyps has been reported after polyp removal by endoscopic ablation, resulting in recurrent anemia [16]. Therefore, wide surgical removal of the polyp was performed in the present case.

## 4. Conclusions

In conclusion, the significance of gastric hyperplastic polyps in gastrointestinal blood loss has not been reported in veterinary medicine. If the gastric polyp is suspected to be the only reason for severe upper gastrointestinal hemorrhage, gastric hyperplastic polyps should be considered in the differential diagnosis of cases presenting with severe anemia.

## Data Availability

Not applicable.

## Supplementary Materials

The following supporting information can be downloaded at: https://www.mdpi.com/article/10.3390/vetsci9120680/s1, Figure S1: Negative result of Warthin-Starry silver staining of the polyp.
