# Supplementary figures and images for: Gastric Hyperplastic Polyp Causing Upper Gastrointestinal Hemorrhage and Severe Anemia in a Dog
Source: Vet Sci. 2022 Dec 7;9(12):680. doi: 10.3390/vetsci9120680 (PMC9782151; doi:10.3390/vetsci9120680)

# Negative result of Warthin-Starry silver staining

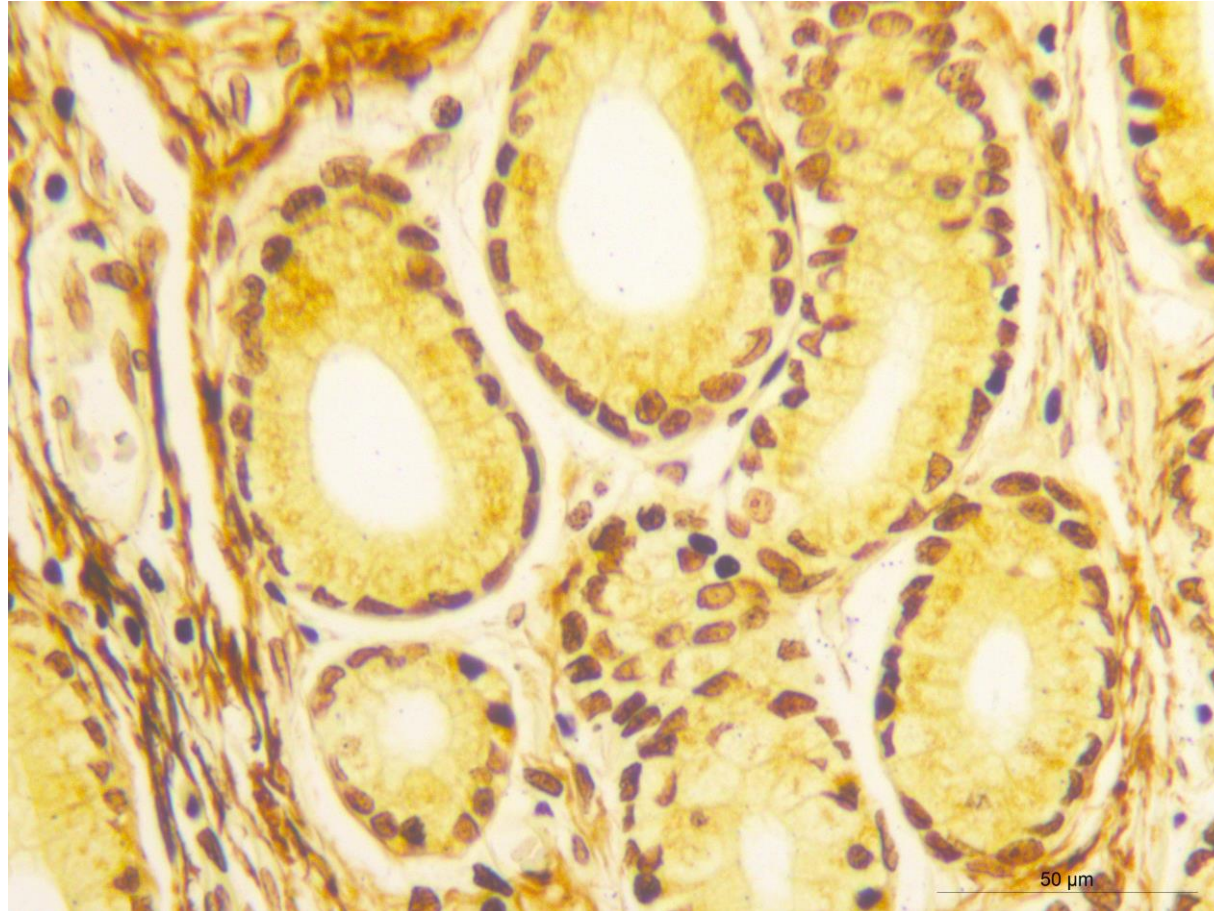

Supplement: Supplementary file 1 [file vetsci-09-00680-s001.zip › vetsci-1917776-Figure S1.pdf]
